# An Effective Barrier to Prevent Crop Contamination by Slug Vectors of *Angiostrongylus cantonensis*

**DOI:** 10.4269/ajtmh.21-1053

**Published:** 2022-04-11

**Authors:** Lorrin Pang, Christy Coppolo, Sara Hauptman

**Affiliations:** Maui District Health Office, Hawaii Department of Health, Wailuku, Hawaii

## Abstract

The accidental ingestion of slugs, intermediate hosts to the *Angiostrongylus cantonensis* parasite, is the most common cause of rat lungworm disease (RLWD) found in humans in Hawaii. This disease has high morbidity and can be complicated to diagnose and treat. With these considerations, efforts in prevention of the initial infection are of high priority. Management of the slug and snail population in food crops is a primary focus to reduce contamination of produce with the rat lungworm (RLW) parasite. The purpose of this study was to prevent RLW crop contamination by preventing the intermediate slug hosts from infesting produce. Our studies showed that an electrified metal tape was a very effective barrier first in the laboratory and then in a garden/farm setting. The intervention is simple to install and maintain and with monitoring for occasional barrier breaches should be able to significantly reduce slug invasion. An integrated pest management program will benefit from the addition of this barrier method to prevent slug carriers of RLWD from infesting produce.

## INTRODUCTION

Rat lungworm disease (RLWD) is a parasitic infection caused by the *Angiostrongylus cantonensis* roundworm that upon ingestion can infect the brain and spinal cord of humans.^1^ This infection can produce neurological symptoms, pain, paralysis, and death and it is one of the principal causes of eosinophilic meningitis.^2^ Rats are the primary host of the *A. cantonensis* parasite, also known as rat lungworm (RLW), which is then transmitted to intermediate slug hosts that eat contaminated rat feces.^2^ Freshwater shrimp, land crabs, frogs, and planarians are considered paratenic hosts of this worm.^2^^–^^5^ Humans are most commonly infected through the deliberate or accidental ingestion of produce infested with slugs carrying RLW.^3^^–^^5^ For the purpose of this paper, the use of the word “slug” refers primarily to land slugs and land snails.

Residents within the state of Hawaii have been affected by an increased incidence of infection over the past several years. The rise in case reports might be because of increased consumption of garden vegetables infested by the recently identified invasive species, *Parmarion martensi* slug (Hawaii and Maui counties), a semi-slug found in high numbers in peridomicile settings.^3^ In a 2004 survey, 40 out of 182 sites evaluated on all six main Hawaiian islands were found to have slugs infected with the RLW parasite. The sites with the highest percentage of slugs infected with RLW were primarily on Kauai and Hawaii at 34% and 33%, respectively, Maui Nui (Maui, Lanai, Molokai) at 18%, and Oahu at 10%.^4^ A more recent gastropod survey focusing on the Island of Maui, in particular, conducted December 2016 to August 2017, found nine species of slugs carrying the RLW parasite.^6^ This study also found that two of the gastropod species were newly identified as carriers of the parasite in Maui and one of the species was new to the state of Hawaii as a whole as a host for the parasite.^6^ It is notable that some slug species, at least currently, have a higher prevalence of parasite infection or carry a higher parasite load than others.^2^

The primary habitat of the slug hosts are in lower elevations with warmer and wetter conditions, thus this is traditionally the location of RLW exposure in humans.^4^ Predictive modeling has indicated that suitable habitats for the RLW parasite will expand by 2100 as it is being found in more native and nonnative gastropod hosts with an increased tolerance for different climates.^4^^,^^7^

Efforts have focused on preventing slugs and snails from coming into contact with food produced in gardens or farms. One suggestion was the installation of barrier materials around the perimeter of garden beds or fields. Some examples included copper tape, mesh, diesel oil, or salt trenches. Another barrier, developed by Chinese escargot (snail) farmers is a plastic “tape” of four metal bands that can be electrified (by battery) for keeping snails contained in escargot “farms.” These four bands with alternating positive and negative DC voltages produce a deterrent shock when adjacent bands are touched simultaneously.

The purpose of this study is to test the effectiveness of the electrified metal tape barrier to deter slugs from entering a farm or garden, thus preventing contamination of produce by potentially RLWD-infected slugs. Small laboratory and field experiments were completed to study the effects of low-voltage shocks as a barrier to slug movement.

## MATERIALS

For both laboratory and field studies, the electric barrier was obtained from China via Alibaba from the Taian Hengtong Plastics Co., Ltd. (electric snail fence tape product).

## METHODS

### Laboratory studies.

Two laboratory studies were conducted in a facility of the Maui Department of Health. The studies were done with the *P. martensi* semi-slugs collected from an area of Maui (Hana) where this species had invaded several years ago. Slugs were collected and delivered to our laboratory and the experiments were run within a day of collection. There were three study groups: the experimental group applying the electrified metal strip and two control groups using either a nonelectrified metal strip or no metal strip.

In the first study, a tower design was set up placing the metal strips around a small cylinder with a solid top and the slugs were placed on top of this cylinder. In order for the slugs to leave, they had to descend across the barriers to get to the bottom of the cylinder. The experiments took place in indirect sunlight to stimulate slugs to move across the barriers.

**Figure 1.**
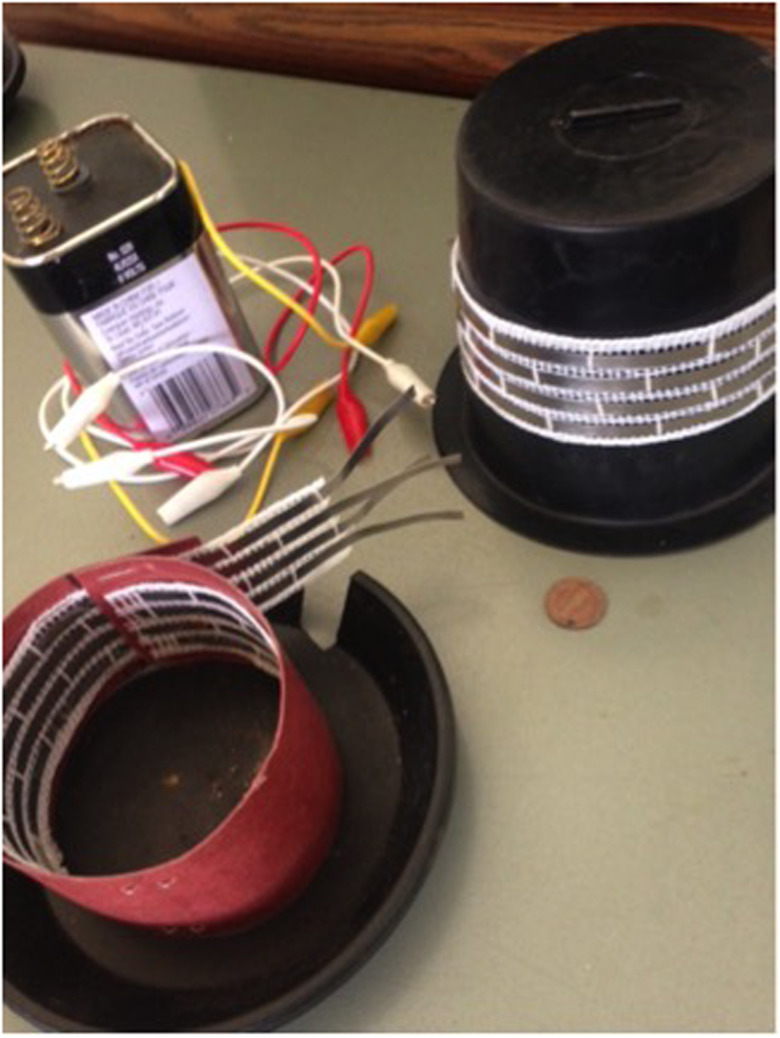
Types of barrier design: Upper right is the tower design used in the first laboratory study. Lower left is the modified design with the barrier tape wrapped on the inside used in the second laboratory study.

The second study used the same three types of barriers design, but placed the slugs inside of a circular container with the barriers wrapped on the inside (Figure 1). Therefore, in order for the slugs to escape, they must climb up across the tape barrier. If they were shocked and fell back into the container, they were still considered a “failure” until they escaped. This study was left to run until any one of the containers emptied of slugs, after which the other two containers were then counted for slugs that remained. Outside of the above protocol procedure, after the study “ended,” the containers were left to run for another 12–24 hours (overnight) at which time slugs were counted in each of the two remaining containers.

### Field studies.

Following the laboratory studies, the barrier assessment moved to the field of a working farm. Here, the goal of the barrier was to prevent slugs from entering crop areas encircled by the barrier of the electrified metal tape. Two field experiments were conducted at a local organic farm in Kula, a town on the island of Maui in Hawaii that provides produce to the local Kula Farmer’s Market. Both field experiments were conducted between May 2020 and February 2021. Slug infestations were identified by surveys and the farmer’s reports of produce infestation and the need for systematic trapping. Although the laboratory studies used *P. martensi* slugs, none were identified in the field; the vast majority of slugs were identified as the slug *Deroceras reticulatum.* The farmer granted full permission for the study staff to access and conduct control efforts during this time period. He also agreed to treat each study site similarly with respect to planting, harvesting, watering, weeding, and no further slug collection or control measures.

**Figure 2.**
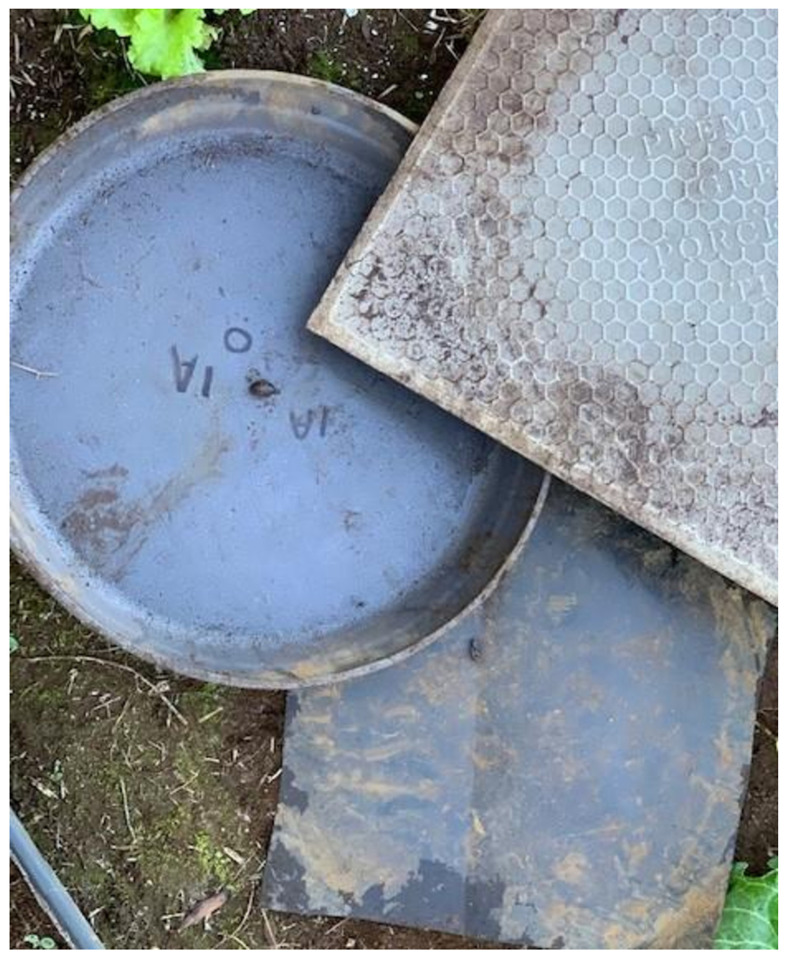
Slug “hut” system consisting of a large planter dish (16″ diameter and 1.5–2″ deep) placed on the ground over an 8″ square sheet of black pond liner and covered with a 16″ square ceramic cover.

To facilitate a systematic, convenient way to count slugs in a designated area, standardized slug “huts” were set up. Each hut consisted of a large plastic planter dish (16″ diameter and 1.5–2″ deep) placed on the ground over an 8″ × 8″ square sheet of black pond liner (20 mil LLDPE [linear low-density polyethylene]) and covered with a 16″ square ceramic cover (Figure 2). After eradication of slugs within each test site, any future slugs found in the site were assumed to have reinvaded the area by climbing over the barriers from outside the site. Slugs were counted in each refuge hut by lifting all layers and counting slugs that were visible, there was no turning over of foliage or dirt. The slugs were not killed or removed, but replaced back under the same hut. This counting method allowed for slug counts per hut and per site to vary (statistically independent) over time. Slugs could leave the sites as well, perhaps less so for the electrified site if they were shocked on exiting. Furthermore, this nonremoval of slugs was done so as not to introduce the *de facto* added “intervention” of slug removal. Therefore, indicating whether the electrified barrier alone worked, farmers would not have to also perform slug removal as an additional form of slug management.

The intent was that these huts would provide cool, humid dark conditions for slugs to gather during the day when systematic counting of slugs could occur. Obviously, not all slugs would be under the huts but these convenience samples would be representative of the slugs in each test site, assuming similar conditions of crop, weed, soil, water, and area conditions. Thus, we assumed that counting slugs under huts would be identically representative (between intervention and control sites) of total slug populations for each site.

**Figure 3.**
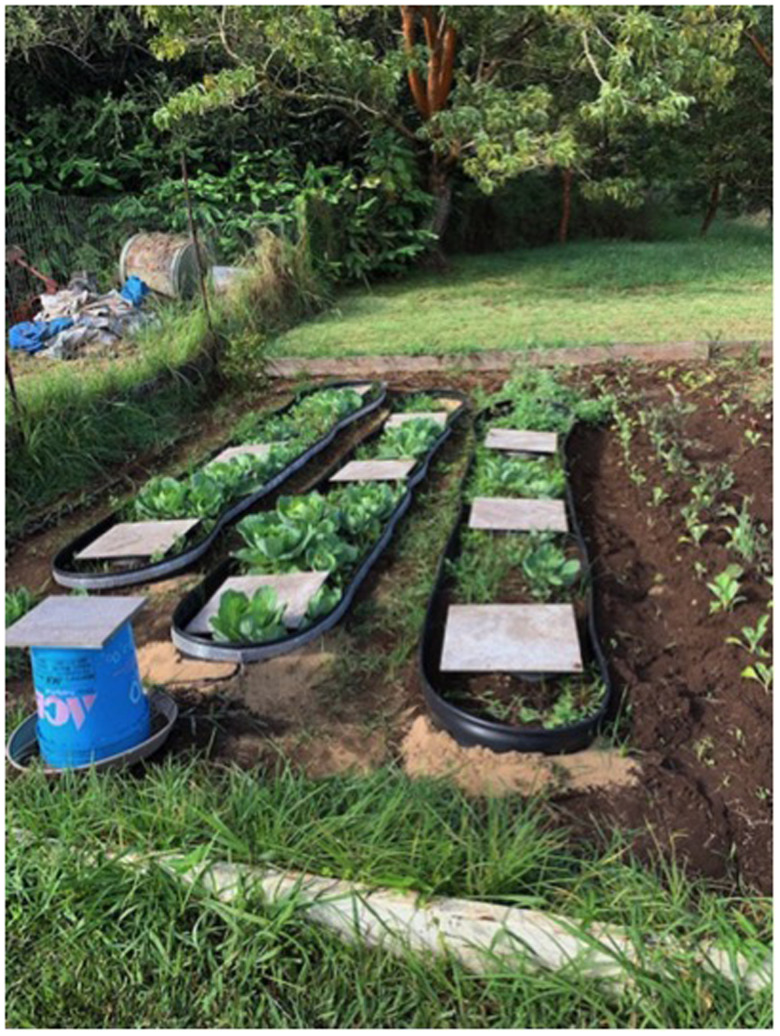
First experiment of the field study with randomized sites consisting of (from left to right): control site 1, experimental site 2, and control site 3 with three slug huts placed within each site.

The first field experiment was conducted from May 27, 2020 to August, 14, 2020. Three adjacent similar sites were selected for the trials, all of the sites were equal in size (about 3’ × 20’) with similar characteristics (soil, water, weather, etc.) including baseline amount of slugs. The randomized sites consisted of the control site 1 enclosed by electric tape that was attached to 5″ plastic lawn edging without electricity, the experimental site 2 enclosed by electric tape that was attached to 5″ plastic lawn edging and electrified using a 6-volt battery, and a second control site 3 enclosed by 5″ plastic lawn edging without the metal tape barrier. The 5″ plastic lawn edging was placed vertically and buried 1 inch under soil to prevent slugs crawling beneath the barrier. Three huts one at each end and one in the middle were placed in each site to standardize the method for slug counts among the three study sites (Figure 3).

At the start of the trial after a 6-volt battery was connected, the sites were eradicated of slugs with the use of organic iron phosphate pellets inside each area on day 0 and again at 2 weeks. This was to ensure all three sites started at the same count as close to zero slugs as possible and to kill slugs (within each site) hatching from eggs during the first two weeks. Live slugs were counted twice a week (semi-week) in each site only under the huts, mainly in the morning. Again, for reasons cited previously, slugs were not removed but replaced under each hut.

The second field experiment was conducted from August 15, 2020 to February 6, 2021 It was similar to the first field experiment except for two changes, based on results of the first experiment. First, only two sites were used; an experimental site with a 6-volt-electrified tape barrier and a control site using the electrical tape barrier without electricity. The second experiment doubled the duration of pellet eradication from 2 to 4 weeks (in both sites), hoping to achieve even lower baseline slug eradication at the start.

For analysis of field experiment data, counts were first graphed over time and examined for periods with consistent effect size (relative counts among sites). Each period was statistically analyzed by χ^2^ (Fisher’s test for rare results) with the null hypothesis of homogeneity comparing counts of control and experimental groups.

## RESULTS

### Laboratory studies.

For the tower study, some slugs “fell” across the barrier instead of crossing the electrified portion. These falling slugs were considered to be “drop outs,” not to be tallied, and the study design was changed to resolve this issue. For the second circular container study, one of the two control containers emptied within an hour. None of the slugs in the experimental group crossed the electrified metal barrier, some were observed (beyond the time limits of the protocol) up to 24 hours with none escaping and eventually all of these slugs died. The results (excluding the results of added observations at 12 and 24 hours) are summarized in Table 1.

**Table 1:** Laboratory results with the total number of slug escapes over three trials (batches of about 9–10 slugs each with controls running concomitantly)

Electrified	Metal	Control (no tape)	
Successful cross	0	25	27
Did not cross	29	4	2
Total	29	29	29

Chi square value = 61.8, *P* < 10^−5^.

### Field studies.

**Figure 4.**
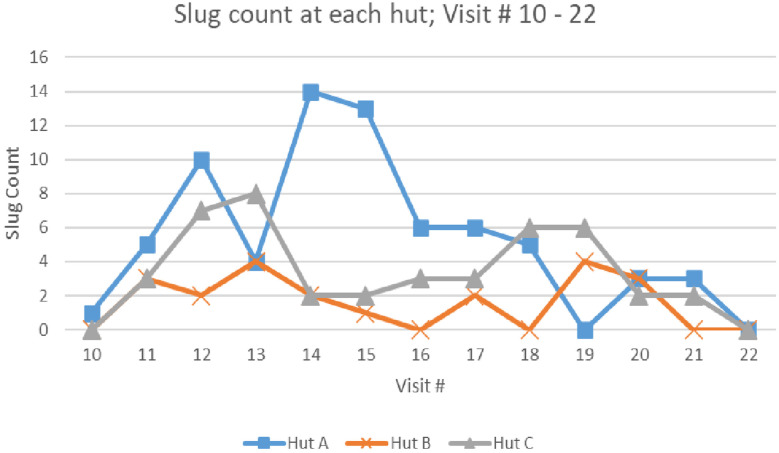
Slug counts over time for each of the three huts (A, B, and C) in one of the control sites during the first experiment of the field study. The horizontal axis is in units of semi weeks.

**Figure 5.**
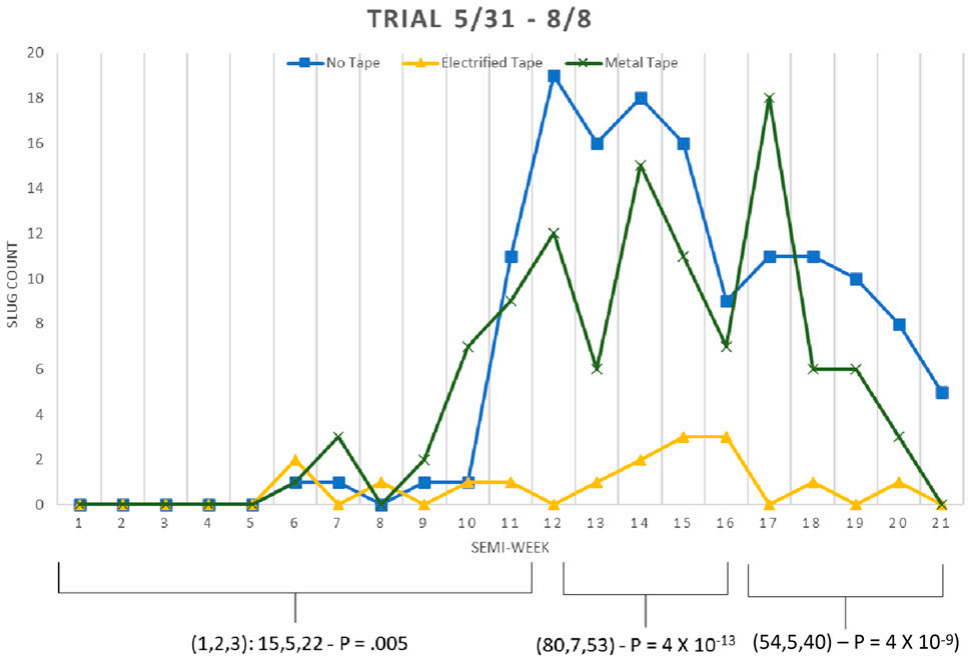
First field study slug counts cumulative for all huts for each site comparing the counts between sites over time. χ^2^ values were calculated under the null hypothesis that all sites had equal counts.

In the first field experiment (the intervention and two controls), Figure 4 shows the slug counts over time for one of the control sites (for each of the three huts A, B, and C). All hut counts are shown to point out that counts vary over time for each hut as well as the total of all huts and that the counts over time are somewhat statistically independent. Figure 5 compares slug counts aggregating the counts across all of the huts for each site over study periods. For example, for the first experiment’s semi-weeks 0–11, the χ^2^ was done on a table of three rows (sites) and 11 columns (semi-weeks). From semi-weeks 0–11, the metal fence, electrified fence, and plastic-only fence had cumulative counts of 15, 5, and 22, respectively (*P* = 0.005); for semi-weeks 12–16, the counts were 87, 7, and 53, respectively (*P* = 4 × 10^−13^); for semi- weeks 17–21, the counts were 54, 5, and 40, respectively (*P* = 4 × 10^−9^). The effect size over all three periods is approximately an 88% reduction in slug counts.

**Figure 6.**
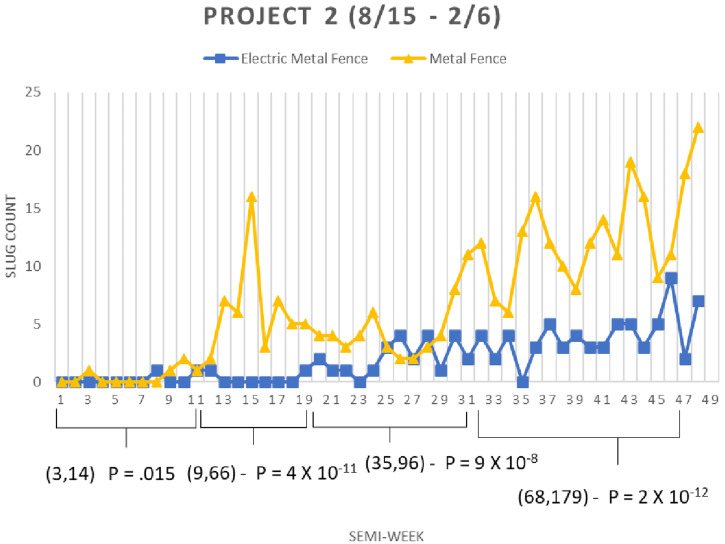
Second field study slug counts cumulative for all huts for each site comparing the counts between sites over time. χ^2^ values were calculated under the null hypothesis that all sites had equal counts.

As in the first experiment, the second experiment aggregates slug counts across all huts for each site comparing the counts over time (Figure 6). For the second experiment from semi-weeks 1–11, the electrified fence and metal fence had counts 3 and 14, respectively (*P* = 0. 015, χ^2^ test for homogeneity); for semi-weeks 11–19, counts were 9 and 66, respectively (*P* = 4 × 10^−11^); for semi-weeks 19–31, counts were 35 and 96, respectively (*P* = 9 × 10^−8^); for semi-weeks 31–45, counts were 68 and 179, respectively (*P* = 2 × 10^−12^). The effect size varies from 86% to 63% reduction in slug counts. Around the time of semi-weeks 19–23, we observed what might be considered potential breaches of the experimental site’s barrier. Cut weeds had piled up on the outside of the barrier perhaps serving as a bridge for slugs to cross over the electric bands. Gaps appeared underneath the plastic support fence probably from irrigation/water erosion. These breaches were fixed and the experiment continued.

There was no significant drop in battery voltage (one 6-volt battery without replacement) throughout both field experiments. Weather and debris appeared to have no effect on the electrical current over time.

## DISCUSSION

Most methods of slug vector control focus on removing and eradicating slugs from crops to minimize crop damage. However, in the case of RLWD, one primary goal should be preventing the invasion of crops by slug vectors. An overall reduction of the prevalence of slugs in food crops could result in a reduction of accidental ingestion of the intermediate hosts to the *A. cantonensis* parasite followed by a reduction of human RLWD.

Farmers have traditionally used many methods to prevent or remove slugs from field produce and studies have shown most of these methods to be ineffective, expensive, and time consuming.^8^ In the very dry areas of Hawaii, slugs might only exist in isolated watered gardens, so periodic eradication may control slugs. However, in many other wetter areas, slugs populate areas surrounding gardens as well, so eradication in garden sites is quickly followed by reinvasion. Some of the slug pellets even have a chemical attractant, bringing in slugs from beyond the boundaries of the treated sites. Under these conditions, effective barriers could prevent this type of reinvasion.

In this study, we showed that an electrified metal tape barrier was successful at reducing the slug population in the laboratory and more importantly in a real-world farm setting. This method was shown to be effective with the *P. martensi* slug in the laboratory and with the *D. reticulatum* (gray field slug) along with common garden snails in the field. There was no discernible difference (compared with controls) in effect of the electrified metal tape barrier on size or age of the slugs. There is no reason to think the electrified barrier would not produce the same results with any slug species as they all have the same basic biological make up and more than likely the same sensitivity to electric shock. If future studies observe significant slug breakthroughs across electrified barriers, then perhaps subgroups (age, size, species, etc.) can be studied.

Although slugs were completely blocked in the laboratory, this setting is somewhat “artificial” since they moved across barriers (or controls) because of aversive stimuli (indirect sunlight). We have observed anecdotally in our laboratory that depending on the slug species, a few slugs will huddle together for hours waiting until darkness, and then move across the nonelectrified barriers overnight. However, in our laboratory setting, no slug ever crossed an electrified barrier day or night. Thus, the field setting better assesses slug movements for both aversion and attractant stimuli, for the controls and the experimental barriers.

Our four experiments, two in the laboratory and two in the field, were designed sequentially to answer issues raised in the preceding experiments. Thus, the separate observations/conclusions complement each other. For example, the field experiments were not 100% effective, whereas the second laboratory experiment was. Beyond the obvious 1) that laboratory and field dealt with different species of slugs and/or 2) that the laboratory used an aversive stimulus to cross the barrier (light), whereas the field attracted them to cross the barrier (scent of vegetation), another explanation might be that in the field, no slugs entered through the barrier but populations hatched inside after the molluscicidal effect of the pellets wore off.

In the second field experiment, we doubled the duration of slug treatment to kill slugs emerging after possible delayed hatching, and did observe a delayed rise in initial slug counts. Unfortunately, this second experiment was marred by possible “breaches”’ to the barrier. Another, subtler, explanation why the field was not 100% effective is that a small percent (hypothetically, suppose 5%) of the slugs actually can withstand the shock and do cross the electric barrier. If this were so (with more slugs initially challenging the barrier from the outside than from within), there would be a rise in cumulative counts over time within the electrified site, until a steady state population (like the controls sites where the number of external and internal challenges are matched) is attained. But rising cumulative counts of the electrified sites were not observed in the field, certainly not to levels in the control sites. The explanation for this could be seen in the first laboratory experiment. Slugs within an electrified site can more easily leave, by falling across the barrier, than enter (again, falling out when shocked). A vertical barrier (electrified on the outside) serves as a partial one-way “valve” resulting in more slugs leaving a site than entering, until a steady state population is reached (with a lower slug prevalence than controls).

Other limitations of the field study included lack of representation of slug species and lack of environmental control. Only the common gray slug, *D. reticulatum*, and common garden snails were found in this particular location in the field study. Further research would be of benefit in other locations, especially locations where the *P. martensi* semi-slug is more predominant as this slug has been found to have a high prevalence of carrying the RLW parasite.^4^

Failure to promptly inspect and repair breaches to the barrier, even for a few days, during the second field experiment could have resulted in a significant slug invasion. The second trial ran for a period of time, beyond (by about 3 months) what the farmer had planned for his crop rotation. Under typical conditions, the produce would have been harvested, the field tilled and replanted. Replacing the electrified barrier and initial slug pellet eradication would also occur at the time of replanting.

Overall, the electrified barrier showed about 90% effectiveness in reducing slug counts in the protected areas. Use of this method will also have the added benefit of reducing the reliance on pesticides, debris removal, and visual surveys. It is recommended that huts be placed strategically within a site to monitor for breaches of the barrier. If identified early, by barrier inspection or rises in slug counts, farmers might be able to respond quickly with repairs and/or reapplication of slug pellets. Although the goal of the electrified metal fence is to deter slugs from entering the site, occasionally slugs were killed when attempting to cross the barrier and their bodies adhered to the metal fencing. The slug bodies were not removed by the investigators and were left to fall off naturally, as part of the “no touch” principle of the study. This was done to see whether there would be a drop in battery voltage (no appreciable drop noted) and to allow other slugs to cross the barrier by climbing over the bodies (but never were there more than two bodies adhering to the entire barrier at any one time). It is recommended to monitor for and remove slug carcasses for the above reasons if this occurs in large numbers.

This is merely a barrier method, however, and as in any integrated pest management program, other methods need to be used along with an efficient barrier. Slugs need to be eradicated at some point when the barrier is electrified. There will also be a need to manage and monitor the field site for breaches of slugs leading to reinvasion; however, this will be significantly less with the use of the barrier method as demonstrated.

Traditionally, slug control methods have focused on using chemicals to kill slugs or baits to trap slugs; however, integrated pest management systems have so far been shown to have the best results.^8^ Evidence collected in our larger field study for the electrified metal tape used on a farm confirmed the effectiveness at controlling slugs in a real-world field setting. The use of the electrified metal tape as a barrier can become the centerpiece in a successfully integrated management program with the primary focus of preventing slug carriers of the *A. cantonensis* parasite from entering protected areas.
